# Gynecological and Obstetrical Society of Baltimore

**Published:** 1894-06

**Authors:** Willim S. Gardner

**Affiliations:** Secretary; 613 Park Avenue


### GYNECOLOGICAL AND OBSTETRICAL SOCIETY OF BALTIMORE.

### SIXTY-FIRST REGULAR MEETING.

*The President, Dr. Thomas A. Ashby, in the chair.*

DR. THOMAS A. OPIE reported three recent abdominal operations.

*Case* 1.—Mrs. B., age forty-five, admitted to the City Hospital, October 13, 1893, suffering from uterine fibroids.

Abdominal hysterectomy was performed October 25th. Patient was discharged November 22, 1894.

From the family history of this case, it appears that a sister died from an ovarian cystoma, which had not been operated on.

We learned from her personal history that she began menstruating at eighteen. From this time she worked hard on her father's farm until twenty-five years of age, when she was married.

After bearing six full term children she ended her faithfulness with a miscarriage. Up to this time she had enjoyed uninterrupted health, save woman's usual embarrassment, constipation.

During five years past, however, her life has been a series of tortures. Dysmenorrhea and menorrhagia were peculiarly distressing at her periods. Severe cramps were realized in the right side of the abdomen, which extended down the right leg. Constipation was ever present. Appetite poor. · Bladder very irritable. She stated that she was confident she often passed her water fifty times a night and when she resisted the prompting during the day or night a deathly nausea came over her. Sexual indulgence was never painful except during a few days preceding the menstrual function.

There was nothing noteworthy in the operation. During four or five days after it, she repudiated every kind of food save small quantities of buttermilk.

The abdominal suture were removed on the ninth day. The urine could not be passed naturally until the thirteenth day. A letter received from her a week since informed me of her enjoyment of excellent health.

I submit the womb which was removed as stated. It serves as an apt illustration of the three forms of uterine fibroids, viz. : Sub-peritoneal, intra-mural and sub-mucous. The first variety is shown in the specimen, at the junction of the body and the neck.

The second is exemplified in numberless small fibroids, scattered throughout the walls of the body and neck of the uterus.

The third is a sub-mucous fibroid tumor, the size of a guinea egg, attached by a pedicle and occupying the cavity of the body of the uterus.

*Case* 2.—M. J., age thirty-seven. Admitted to City Hospital, November 15, 1893.

*Diagnosis :* Large multiple fibroid mass on the left side encroaching upon the liver and a second tumor cystic in character occupying a lower position on the right side of the abdomen. According to the history given us of her case, the catamenia began at thirteen years of age and was regular during seventeen years. At the age of thirty, therefore, she is said to have contracted a severe cold at her period and afterwards had a complete suppression of her flow until one year ago, when severe hemorrhages set in, which lasted on an average three weeks at a time. The patient had noticed an abdominal enlargement for the past six years, which gradually grew larger. In the meanwhile, when at thirty-three years of age (four years ago), she married. During the last four months she has been a sufferer from nausea, diarrhea and dyspepsia. Her size has been that of the full term of pregnancy. The operation of supra-abdominal hysterectomy was performed November 17, 1893. The abdominal incision having been made and the fibroid and cystic elements having been confirmed, the patient was turned to the left side, the cyst was opened with the knife, when the flow of amniotic fluid and blood announced the fact of pregnancy. The operation was proceeded with. There was no hemorrhage. The pedicle gave no trouble; indeed, it presented the neatest appearance after closure of the flaps and all betokened as good a result as any case of hysterectomy I had ever performed. The fetal development showed the pregnancy to be about four and one-half months advanced. On the day following the operation the patient had rallied well, her pulse was steady and strong and the temperature normal. November 19th slight jaundice supervened. Emesis occurred but not troublesome. November 20th the jaundice had markedly increased, but pulse was rapid and weak; expression anxious and vomiting continuous. Death ensued at 11 P. M., forty-six hours after the operation.

The kidneys and liver presented at the post-mortem, held by Professor Kierle, marked fatty change. The left lobe, which had been under pressure by the fibroid mass on the left side, was pale and anemic, giving strong contrast with the right lobe, neither being normal. Was this an attack of acute septicemia or was there obstructive jaundice here, which brought about the fatal result?

*Case* 3.—B. C. S., age eleven years; a schoolgirl. Was admitted to Baltimore City Hospital. Her parents first observed an enlargement of the abdomen more than four and a half years ago. There is no history of menstruation, nor has she ever had an accident or serious illness. She has always enjoyed good general health, though she has not been able to study hard or regularly at school. For the last three months she has complained of pain in her (stomach) abdomen. During two years she has had some headache and dizziness. Tapping had been resorted to twice. Each time one-half ounce of yellow fluid is said to have been withdrawn.

When first examined by Drs. Thomas, Bailey, Chambers and myself, the abdomen was distended by a large, fluctuating tumor, which completely filled up the abdominal cavity. The prevailing opinion of the examiners was that the case was either hydatids of the liver, ovarian cystoma or a kidney cyst.

When the patient was upon the operating table in the dorsal decubitus and under anesthesia, by partially raising her with the hand under her back so that only the shoulder and sacrum rested on the table, it was perceptible that the tumor was not a pelvic one, since it gravitated away from this region. Moreover, percussion gave a continuity of dullness over the liver and the tumor. Upon opening the abdomen a large unilocular cyst was found resembling an ovarian cystoma both as to the wall and fluid contents. It was attached to the base of the liver between the gall-bladder which was incorporated in the sack wall on the left side and the spigelu, caudate and right lobes. No definite pedicle could be made out. The sack was punctured and the contents, all of which were fluid, emptied, and the sac stitched into the upper part of the wound, the superfluous portion having been cut away. A large gauze drain was inserted along the track of the collapsed sac. It has now been over six weeks since the operation was performed. The wound is still open and the probe can be inserted up to the attachment of the sac to the liver. There is still a continuous, clear discharge, amounting to about four ounces in twenty-four hours. The child's general improvement is very pronounced. Her mother states that she has never known her to look so well. Nevertheless, the sac, which has been constantly washed out with the bichloride and a solution containing equal parts of tincture iodine and acid carbolic, continues to elaborate its secretion.

Professor Kierle found Drysdale's corpuscles in the fluid taken at the time of the operation from the sac.

DR. B. B. BROWNE mentioned a case in which the child was delivered by a mid-wife. Subsequent examination by Dr. Browne showed the presence of a large sized fibroid, but as involution progressed the tumor became smaller until only a small knot remained.

DR. T. A. ASHBY mentioned a case in his own experience in which a woman aborted several times, this being apparently due to the pressure of a fibroid. Dr. Ashby also showed a uterus with a multiple fibroid which he had removed that morning.

DR. J. EDWIN MICHAEL reported the following case of sudden death after labor:

Mary A., colored, age twenty-eight, was in labor when admitted Upon examination child was found to be transverse. The os was fully dilated, membranes intact and patient was in fair condition. No history as to how long patient had been in labor. The contractions of the uterus were hard and long and came on at intervals of three or four minutes. She was admitted at 4:30 A. M., and was allowed to go on until 7 A. M., when it was decided to do internal podalic version. Patient was put to sleep with chloroform, membranes ruptured and a large amount of amniotic fluid escaped. Patient put in an exaggerated lithotomy position, right hand of operator introduced and came in contact with hands of fetus, which were pushed up out of the way. Hand passed to left and breach felt and the left or anterior leg caught and brought down to os. Right leg could not be reached with right hand, so left was introduced and passed along posterior uterine wall and right leg found folded back on dorsum of child. It was brought down and then both feet were pulled through vulva orifice.

Owing to the slippery condition of the child's feet and exhaustion of the operator, sufficient traction could not be made to turn the child. A piece of bandage was passed around left foot and traction made and child turned easily, giving the position of R. S. I. A. Steady traction was then made and breach easily delivered. Both hands were caught up over the head and were delivered with some difficulty. Head delivered by Veit-Smellie method with some difficulty. Uterus contracted well and expelled the placenta spontaneously. Operation occupied one hour. Patient seemed in fair condition, with pulse and respiration good. While vaginal douche was being given, patient suddenly lost pulse at the wrist and respiration slowed. The anesthetic was stopped and three hypodermics of whisky given which improved her. Next uterine douche was given and foot of bed elevated. Hypodermic of strychnia sulphate, grs, $\frac{1}{30}$, ergotole m. xxv., hypodermically, this followed by a hypodermic of $\frac{1}{100}$ gr. of nitro-glycerine and whisky. Limbs bandaged to hips. Hot bottles applied. Patient now being conscious was given hot coffee and whisky every fifteen minutes. Patient then moved to another bed and another hypodermic of nitro-glycerine $\frac{1}{100}$ gr.

Whisky and strychnia sulphate grs. $\frac{1}{50}$ was also given. Later hypodermic of ergotole m. xxv. Patient complained of pain in the legs and a want of air and wanted to turn on her left side. Pulse became slower and weaker, respiration less frequent, patient more pallid and died at 9:35 A. M., one hour and thirty-five minutes after the operation. Post-mortem showed uterus intact, kidneys white and fatty, intestines bleached and lungs pale.

Baby was dead when delivered and weighed nine pounds and twelve ounces.

This is the first case of the kind I have seen ; they are quite rare. The post-mortem was of especial interest.

Dr. Lawson, who did the work, is an experienced and competent man. I saw the case half an hour after the version was done. Everything was done that we thought could be of use to restore her. I was afraid that rupture of the uterus had occurred, but the post-mortem showed that it did not take place. The lungs were pale and bloodless, heart contracted, blood vessels contracted and empty ; the conditions justified the conclusion of death by shock. There are cases in surgery in which there is shock severe enough to produce death where the traumatism is nothing near so great as in labor. A number of cases have been recorded where the passage of a sound has caused death by shock. So the surprise is that we do not have more cases of death from shock.

Eight causes of sudden death after labor are mentioned by Parvin in a paper on that subject.

I. Mental shock.
II. Result of severe pain.
III. Diseases of the heart.
  *a.* Laceration.
  *b.* Paralysis from over distention.
IV. Diseases of blood vessels.
  *a.* Aorta, or
  *b.* Splenic vessels.
V. Accidents of labor.
  *a.* Post-partum hemorrhage.
  *b.* Rupture of uterus.
VI. Pulmonary thrombosis.
VII. Air embolism.
VIII. Respirative difficulties due to diseases of the lung or pressure on the lungs.

These eight heads do not fully cover the ground. As I said in the beginning we have the conditions of shock in obstetrics as well as in surgery. And traumatism of the genital apparatus, both in male and female, seems specially liable to produce severe shock.

DR. THOMAS A. ASHBY: Dr. Riley called for me recently to see a case of a patient who had lost very little blood, but she was in such profound shock that I thought she would not live ten minutes. It was the most profound shock I have ever seen. Abundance of whisky and nitro-glycerine were all that rescued her. There was no lesion detected, only the shock from delivery.

---

SIXTY-SECOND MEETING.

*The President, Dr. T. A. Ashby, in the chair.*

DR. G. LANE TANEYHILL read a paper on "Puerperal Mania" in which he gave his personal experience. He confined his paper to that division termed by Lusk "The Insanity of Childbed" and by Ramsbotham that form attended by great excitement and furious delirium, intentionally ignoring the other two forms, the "Insanity of Pregnancy" and the "Insanity of Lactation."

When we consider how active are the causes of physical disturbances in women during their child-bearing period, we need not be astonished at Tuke's assertion that of the insane admitted to asylums, *in one eighth* the affection is of puerperal origin. Of the seven cases he had treated, five were of the excitable furious form; this form predominates in Burrows' statistics, who, out of a total of fifty-seven, noted thirty-three as maniacal, sixteen as melancholic and eight as alternating, and Playfair gives the relative proportions of each form, per hundred as follows:

| | |
|---|---|
| Insanity of pregnancy | 18 per cent. |
| Puerperal insanity | 47 per cent. |
| Insanity of lactation | 34½ per cent. |

Women endowed with acute feelings of an excitable temperament, and especially unmarried women who have conceived and who experience a deep mortification and have necessarily been exposed to other harmful factors, are probably more predisposed to this affection than others. Of ninety-two cases treated by Esquirol twenty-nine were "illegitimately pregnant."

Many causes are assigned for this sad affliction, among which is "that undue excitability of the nervous system present during pregnancy, labor, and the chief portion of the process of lactation." Half of the eighty lying-in women who were treated by Burrows and who became delirious had an hereditary disposition to insanity. Terror and alarm had been assigned as causes in two of Dr. Taneyhill's cases.

Diseases antedating pregnancy, hemorrhages and eclampsia may be considered proximate but not fundamental causes of puerperal mania. Septicemia is mentioned by Sir James Simpson as a cause in four patients in whom he found albumen in the urine, and Dr. Donkin, in the Seventh Volume *Edinburgh Medical Journal*, sustains the view calling it renal puerperal mania.

Playfair, however, criticises this hypothesis by asserting that the albuminuria is transient, while its supposed effects last for months, and says why should uremic poisoning in one case cause insanity, and in another convulsions.

The impatient, irritable, suspicious victim of this malady may first fill the attendants with consternation by suddenly breaking out in a wild torrent of invective against her husband or some dear relative. This will frequently be followed by listlessness, obstinacy and an absolute ignoring of the fact that she has lately given birth to a child, or she may insist that the child has died or is that of another.

The countenance is changed, the sweet disposition becomes one of studied revenge, the chaste demeanor is supplanted by immorality and obscenity of language incredible except to those who have been compelled to be in the presence of such a maniac; inattention and perverseness will follow in the quieter intervals. Frequently the lochia are suppressed and bowels constipated and persistent insomnia continues to resist some of the most approved anodynes.

Gooch, in his work on "Disorders of the Mind in *Lying-in Women*," refers to a case of delirium tremens which simulated this disease, but the known habit of indulgence, or the contrary, in a few days reveals the true nature of the case. Phrenitis, encephalitis, or acute delirium, although like puerperal mania, are accompanied by violent and furious excitement, yet we also have tinnitus, vertigo, severe pain in the head, high fever, hard pulse, congested eyes and intolerance of light; whereas in puerperal mania we generally have a quick but soft pulse, seldom any rise of temperature, face pale and impressing one as if the patient were suspicious of some betrayal or calamity about to ensue, with eyes bloodless and without expression, and a disposition to gaze contentedly at the mid-day sun. After relating several amusing cases of mistaken delirium tremens and phrenitis for puerperal mania, he referred to the prognosis, remarking that his fatal cases were those occurring soon after delivery and having a continuously rapid pulse, two out of seven having died. Esquirol, in treating ninety-two cases in four years, had fifty-five recoveries, and Burrows reported from fifty-seven cases, thirty-five recoveries, eleven remaining uncured, ten dying and one committing suicide. Thus it will appear that Lusk is safe in estimating the recoveries at sixty per cent.

He had seen but one post mortem of a subject of this distressing malady, and the only discovery of note was a marked absence of blood in the brain; he knew of no other post mortem condition peculiar to the disease. Restraint and seclusion he considered absolutely necessary in the treatment; send her to an institution, if possible one on the "cottage plan," undisturbed by the noise of other maniacs and withdrawn from the visits of friends and relatives. If compelled to treat her at her own house take a high quiet room; ventilate, but screw sash to window frames, remove "dangerous" articles of furniture, for these maniacs are suicidal; interdict visits and retain a patient, intelligent, matronly nurse. "Combat morbid symptoms as they arise," allay nervous excitement but abstain from any medication that may exhaust the patient; *freely evacuate the bowels*, restore the lochia if suppressed, freely use the warm sponge bath, and of all things secure for her an abundance of *sleep;* he gives half a grain of morphia hypodermically each night, repeating it in six hours if necessary. As "excitation is not inflammation," and as Burrows says muscular exertion is not vital power, we should not in these cases resort to blood-letting and powerful sedatives. If the action of morphia resisted, Dr. Taneyhill resorted to thirty grain injections of hydrate chloral per rectum in warm milk. She should be compelled to take a free and full diet to counteract the astonishing waste of tissue whcih supervenes.

In 1868, as we discharged a beautiful young married woman from the Maryland Hospital for the Insane, sound in mind and body, he remarked to the Superintendent that it was sad to think that she had any ovaries, implying, of course, that she should not again be subjected to the liability of conception.

It is said that Professor Goodell, at International Medical Con gress of 1881, remarked that every insane woman should be de prived of her ovaries; he was not prepared to express such a radical opinion, but had observed in the *American Journal of Obstetrics*, 1892, that Dr. Rohe of this Society, had the courage, in a moderate number of cases, to bring to the test of experience this hypothesis.

Four of the cases operated on were those of puerperal mania. We are told that two were improved, but not cured, and two left the asylum quite well, a few months after the operation. A conservative man might venture the opinion that at least in cases of recurrent puerperal mania where the sexual disorder is clearly responsible for the insanity, the ovaries ought to go.

In convalescence give your patient a change of location and air, and from this, with new environment and the constant presence of an intelligent, cherry nurse, and yet with few visits for several months by relatives, we may reasonably expect complete restoration.

DR. ROHE: I want to take occasion to say that I do not agree with the opinion of Dr. Goodell expressed in 1881, or his present opinion, which is just the opposite. The general consension of opinion is that in the majority of instances puerperal insanity is due to septic infection.

I think that opium in any maniacal condition, unless necessary to maintain strength, is bad. Chloral is much better, unless the heart is in bad condition, and in these cases it can be combined with digitalis. Assuming that most cases of puerperal mania are due to sepsis, and that opium is bad in septic conditions, I think opium is bad in this disease. Chloral, with digitalis, or sulphonal, or trional, is better.

DR. NEALE reported the following case of puerperal mania: Mrs. D., white, thirty-eight years, primipara, delicate, nervous temperament, and probably tuberculous. Family history of insanity only on father's side. Patient had recently been under gynecological treatment, and complained of a fistula discharging into vaginal entrance, the orifice of which I could not find at my first and only examination made before confinement.

Was summoned to attend her in labor at term during afternoon of May 27, 1885. Pains at first scarcely appreciable, gradually increased and the slow tedious labor was terminated naturally at 12:20 P. M., May 29, 1885. Slight perineal laceration sustained. After labor patient continued very restless, complained of pain in chest and abdomen, vomited and did not sleep until four one-fourth grain doses of morphia sulphate had been given hypodermically at intervals of one hour. She was delivered at 12:20 P. M. and at 10 P. M. temperature was $100\frac{1}{5}$, pulse 112, respiration 24. After a restless night, I found patient next morning, May 30th, with temperature 102, pulse 112, respiration 24, and complaining of pain in chest and abdomen, for which no local cause could be found.

MANIACAL ATTACK.

At 2 P. M., May 30th, nearly thirty-six hours after delivery, patient screamed out with pain in chest and abdomen and also loudly shrieking "my back is breaking," and became violently maniacal, voiding urine freely in bed. I at once gave her ten minims of Magendie's solution hypodermically, which was followed by sleep. Upon immediate consultation with Professor Miltenberger, puerperal septicemia complicated by mania was diagnosed, and the patient was given twenty grains of chloral every two hours according to effect produced. Sleep followed throughout most of the night, but she was maniacal whenever awake.

May 31st A. M., temperature $100\frac{2}{5}$ pulse 112, respiration 24. Patient conscious and better. Mania recurred during the morning, however, slight tympanites developed, lochia became scanty and tainted, and temperature and pulse gradually increased. The uterus was washed out, quinine was given internally, together with liberal stimulation and morphine according to mania, sleeplessness, etc., but she sank and died at 6 A. M., June 1, 1885.

DR. J. EDWIN MICHAEL: I have seen only one case of puerperal insanity; it was due to sepsis, had symptoms of melancholia and finally recovered. She was treated with bromidia. I agree that these cases are associated very often with sepsis but that does not account for all of them; heredity is no doubt a very powerful causative agent. We should remember that women who have a hereditary taint may be attacked during the puerperal period.

DR. WILMER BRINTON: I have seen three cases; all went to institutions for the insane, all died. One case had mania in her first confinement and recovered. With her second child the mania returned and she died. In another case the insanity came on the fourteenth day; she was treated at home for some time, and at last was sent to an asylum, where she died in three or four weeks. While I agree with Dr. Rohe that chloral and bromide are better than opium in most cases, there are exceptional cases where the opium is much better.

DR. JOHN NEFF: I have had three cases of puerperal mania. The first patient was Irish, the labor normal; the puerperal period was normal up to the fifth day, when her husband came home drunk, and in twenty-four hours she had developed puerperal mania. She was removed to an asylum and died in five days.

The second case had eclampsia which came on before and continued twenty-four hours after labor. She developed puerperal mania, from which she recovered at home. She has since been confined and has had neither eclampsia nor insanity.

The third case was treated at home without success and afterward sent to a private asylum, where she apparently recovered. During the following five years she was well most of the time, but finally committed suicide.

DR. ASHBY: I have seen only one case of puerperal insanity, and that not a violent one. There was a bad family, history and she had had slight attacks before she was confined. She has recovered, but has not been perfectly sound.

I have had two cases after laparotomy in which I consider sepsis to be the direct cause.

In one case seven days after operation pus collected in the pelvis, and she became wild and maniacal. Three weeks afterward the sepsis cleared up and she recovered.

The second case occurred recently. I removed a large pus sac, which ruptured, and I used drainage. At the end of seventy-two hours she developed mental trouble, jumped out of the bed, tore open the wound, but finally recovered without a rise of temperature above one hundred degrees. When the wound was completely healed the mental trouble disappeared.

DR. G. LANE TANEYHILL, in closing the debate, remarked that he *had* mentioned septicemia as a cause, but did not dwell on it in speaking of treatment, for none of his seven cases were traceable to septicemia. Four recovered, two died and one went into profound melancholy. He did not agree with the modern gynecologists, that nearly all the cases of puerperal mania are attributable to septicemia. He could even in these days administer morphia hypodermically in large doses to the raving puerperal maniac in preference to giving sulphonal or paraldehyde. Yes, he proposed, and did use, "mechanical restraint" in certain cases of this disease, when the wild woman, after a struggle of four hours of excitement, was not bodily controlled by the nurses and continued to resist strong anodynes administered in different ways. It must be remembered his paper exclusively contemplated those cases characterized by furious delirium.

WILLIM S. GARDNER, M.D.,
613 *Park Avenue.* Secretary.

